# (*Z*)-*N*-(3-Nicotinoyl-1,3-thia­zolidin-2-yl­idene)cyanamide

**DOI:** 10.1107/S1600536810014406

**Published:** 2010-04-24

**Authors:** Yun-Man Xie, Yu-Min Li

**Affiliations:** a Henan Chemical Industry Research Institute Co Ltd, Zhengzhou 450052, People’s Republic of China

## Abstract

In the title compound, C_10_H_8_N_4_OS, the dihedral angle between the pyridine and thia­zolidine rings is 52.5 (5)°. Inter­molecular C—H⋯N inter­actions help to stabilize the crystal structure.

## Related literature

For related structures, see: Wang *et al.* (2008 ▶); Liu & Li (2009 ▶). For the biological activity of thia­zolidine-containing compounds, see: Iwata *et al.* (1988 ▶); Ogawa (2000 ▶). For bond-length data, see: Allen *et al.* (1987 ▶).
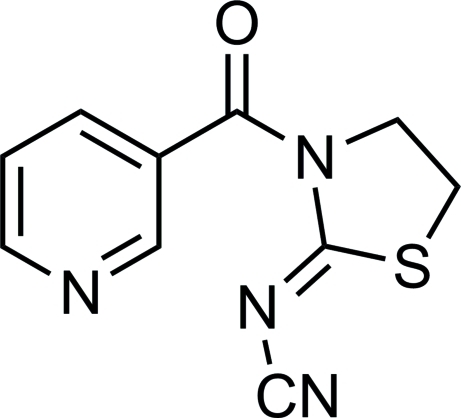


## Experimental

### 

#### Crystal data

C_10_H_8_N_4_OS
*M* *_r_* = 232.26
Monoclinic, 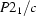

*a* = 5.9180 (12) Å
*b* = 15.182 (3) Å
*c* = 11.448 (2) Å
β = 94.62 (3)°
*V* = 1025.2 (4) Å^3^
*Z* = 4
Mo *K*α radiation
μ = 0.30 mm^−1^
*T* = 173 K
0.17 × 0.07 × 0.05 mm

#### Data collection

Rigaku Mercury CCD/AFC diffractometer
Absorption correction: multi-scan (*CrystalClear*; Rigaku, 2007 ▶) *T*_min_ = 0.951, *T*_max_ = 0.985
7491 measured reflections
1799 independent reflections
1699 reflections with *I* > 2σ(*I*)
*R*_int_ = 0.054

#### Refinement

*R*[*F* ^2^ > 2σ(*F* ^2^)] = 0.043
*wR*(*F* ^2^) = 0.095
*S* = 1.15
1799 reflections
145 parameters
H-atom parameters constrained
Δρ_max_ = 0.28 e Å^−3^
Δρ_min_ = −0.19 e Å^−3^

### 

Data collection: *CrystalClear* (Rigaku, 2007 ▶); cell refinement: *CrystalClear*; data reduction: *CrystalClear*; program(s) used to solve structure: *SHELXS97* (Sheldrick, 2008 ▶); program(s) used to refine structure: *SHELXL97* (Sheldrick, 2008 ▶); molecular graphics: *SHELXTL* (Sheldrick, 2008 ▶); software used to prepare material for publication: *SHELXTL*.

## Supplementary Material

Crystal structure: contains datablocks I, global. DOI: 10.1107/S1600536810014406/hg2674sup1.cif

Structure factors: contains datablocks I. DOI: 10.1107/S1600536810014406/hg2674Isup2.hkl

Additional supplementary materials:  crystallographic information; 3D view; checkCIF report

## Figures and Tables

**Table 1:** Hydrogen-bond geometry (Å, °)

*D*—H⋯*A*	*D*—H	H⋯*A*	*D*⋯*A*	*D*—H⋯*A*
C2—H2*A*⋯N4^i^	0.93	2.52	3.383 (3)	154
C8—H8*B*⋯N1^ii^	0.97	2.55	3.481 (3)	162

Symmetry codes: (i) 
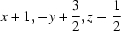
; (ii) 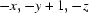.

**Fig. 1.**
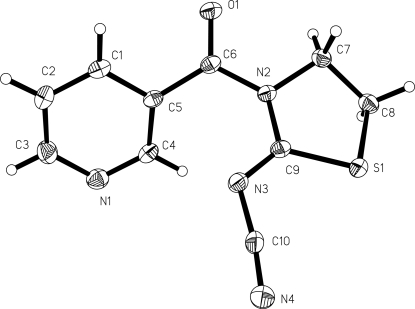
The molecular structure of (I), with atom labels and 40% probability displacement ellipsoids for non-H atoms.

## supplementary crystallographic information

### Comment

Thiazolidine is an important group in organic chemistry. Many compounds containing thiazolidine groups possess a broad spectrum of biological activities (Iwata *et al.*, 1988; Ogawa, 2000). In order to search for new thiazolidine compounds with higher bioactivity, we synthesized the title compound and describe its structure here.

In title compound, all bond lengths in the molecular are normal (Allen *et al.*, 1987) and in a good agreement with those reported previously (Wang *et al.*, 2008; Liu & Li, 2009). The dihedral angle between pyridine (C1—C5/N1) and thiazolidine (C7—C9/N2/S1) rings is 52.5 (5)°. The intermolecular C—H···N hydrogen bonds stabilize the structure.

### Experimental

A mixture of *N*-cyanoiminothiazolidine 10 mmol (1.27 g), nicotinoyl chloride (1.42 g, 10 mmol) and (1.01 g, 10 mmol ) triethylamine is refluxed in absolute acetone (25 ml) for 4 h. On cooling, the product crystallizes and is filtered, and recrystallized from absolute EtOH, yield 2.13 g (92%). Single crystals suitable for X-ray measurements were obtained by recrystallization from dichloromethane at room temperature.

### Refinement

H atoms were positioned geometrically and refined using a riding model, with C—H = 0.93 or 0.97 Å and with *U*_iso_(H) = 1.2 times *U*_eq_(C).

### Crystal data

**Table:**

C_10_H_8_N_4_OS	*F*(000) = 480
*M**_r_* = 232.26	*D*_x_ = 1.505 Mg m^−^^3^
Monoclinic, *P*2_1_/*c*	Mo *K*α radiation, λ = 0.71073 Å
Hall symbol: -P 2ybc	Cell parameters from 3351 reflections
*a* = 5.9180 (12) Å	θ = 1.3–27.5°
*b* = 15.182 (3) Å	µ = 0.30 mm^−^^1^
*c* = 11.448 (2) Å	*T* = 173 K
β = 94.62 (3)°	Needle, colorless
*V* = 1025.2 (4) Å^3^	0.17 × 0.07 × 0.05 mm
*Z* = 4	

### Data collection

**Table:**

Rigaku Mercury CCD/AFC diffractometer	1799 independent reflections
Radiation source: Sealed Tube	1699 reflections with *I* > 2σ(*I*)
Graphite Monochromator	*R*_int_ = 0.054
φ and ω scans	θ_max_ = 25.0°, θ_min_ = 2.2°
Absorption correction: multi-scan (*CrystalClear*; Rigaku, 2007)	*h* = −6→7
*T*_min_ = 0.951, *T*_max_ = 0.985	*k* = −18→18
7491 measured reflections	*l* = −13→13

### Refinement

**Table:**

Refinement on *F*^2^	Primary atom site location: structure-invariant direct methods
Least-squares matrix: full	Secondary atom site location: difference Fourier map
*R*[*F*^2^ > 2σ(*F*^2^)] = 0.043	Hydrogen site location: inferred from neighbouring sites
*wR*(*F*^2^) = 0.095	H-atom parameters constrained
*S* = 1.15	*w* = 1/[σ^2^(*F*_o_^2^) + (0.0269*P*)^2^ + 0.7053*P*] where *P* = (*F*_o_^2^ + 2*F*_c_^2^)/3
1799 reflections	(Δ/σ)_max_ = 0.001
145 parameters	Δρ_max_ = 0.28 e Å^−^^3^
0 restraints	Δρ_min_ = −0.19 e Å^−^^3^

### Special details

**Table:**

Geometry. All esds (except the esd in the dihedral angle between two l.s. planes) are estimated using the full covariance matrix. The cell esds are taken into account individually in the estimation of esds in distances, angles and torsion angles; correlations between esds in cell parameters are only used when they are defined by crystal symmetry. An approximate (isotropic) treatment of cell esds is used for estimating esds involving l.s. planes.
Refinement. Refinement of *F*^2^ against ALL reflections. The weighted *R*-factor wR and goodness of fit *S* are based on *F*^2^, conventional *R*-factors *R* are based on *F*, with *F* set to zero for negative *F*^2^. The threshold expression of *F*^2^ > σ(*F*^2^) is used only for calculating *R*-factors(gt) etc. and is not relevant to the choice of reflections for refinement. *R*-factors based on *F*^2^ are statistically about twice as large as those based on *F*, and *R*- factors based on ALL data will be even larger.

### Fractional atomic coordinates and isotropic or equivalent isotropic displacement parameters (Å^2^)

**Table:**

	*x*	*y*	*z*	*U*_iso_*/*U*_eq_	
S1	0.07382 (9)	0.71271 (4)	0.18314 (5)	0.02782 (18)	
O1	0.6823 (2)	0.58746 (10)	−0.01165 (14)	0.0305 (4)	
N1	0.0795 (3)	0.54439 (13)	−0.29129 (17)	0.0316 (5)	
N2	0.3613 (3)	0.63953 (12)	0.05512 (15)	0.0230 (4)	
N3	0.0875 (3)	0.72532 (12)	−0.05117 (15)	0.0253 (4)	
N4	−0.2685 (4)	0.81222 (15)	−0.06511 (18)	0.0426 (6)	
C1	0.5188 (4)	0.60983 (14)	−0.2489 (2)	0.0264 (5)	
H1A	0.6663	0.6306	−0.2347	0.032*	
C2	0.4329 (4)	0.58936 (16)	−0.3616 (2)	0.0318 (5)	
H2A	0.5202	0.5972	−0.4248	0.038*	
C3	0.2154 (4)	0.55716 (16)	−0.3780 (2)	0.0338 (6)	
H3B	0.1591	0.5434	−0.4540	0.041*	
C4	0.1635 (4)	0.56544 (14)	−0.1829 (2)	0.0262 (5)	
H4A	0.0720	0.5573	−0.1214	0.031*	
C5	0.3806 (3)	0.59877 (13)	−0.15738 (19)	0.0217 (5)	
C6	0.4871 (4)	0.60973 (13)	−0.03604 (19)	0.0228 (5)	
C7	0.4663 (4)	0.63253 (15)	0.17646 (18)	0.0254 (5)	
H7A	0.5699	0.6811	0.1940	0.030*	
H7B	0.5496	0.5777	0.1871	0.030*	
C8	0.2732 (4)	0.63538 (15)	0.25500 (19)	0.0268 (5)	
H8A	0.3252	0.6557	0.3329	0.032*	
H8B	0.2048	0.5777	0.2611	0.032*	
C9	0.1716 (3)	0.69261 (14)	0.04735 (19)	0.0222 (5)	
C10	−0.1045 (4)	0.77156 (15)	−0.05189 (18)	0.0280 (5)	

### Atomic displacement parameters (Å^2^)

**Table:**

	*U*^11^	*U*^22^	*U*^33^	*U*^12^	*U*^13^	*U*^23^
S1	0.0233 (3)	0.0389 (4)	0.0215 (3)	0.0052 (2)	0.0034 (2)	−0.0013 (2)
O1	0.0204 (9)	0.0388 (9)	0.0324 (9)	0.0057 (7)	0.0024 (6)	0.0012 (7)
N1	0.0276 (11)	0.0311 (11)	0.0363 (12)	−0.0026 (8)	0.0029 (9)	−0.0036 (9)
N2	0.0185 (9)	0.0278 (10)	0.0228 (10)	0.0038 (7)	0.0023 (7)	0.0007 (8)
N3	0.0237 (10)	0.0293 (10)	0.0232 (10)	0.0055 (8)	0.0044 (7)	0.0029 (8)
N4	0.0399 (13)	0.0566 (14)	0.0306 (12)	0.0207 (12)	−0.0015 (9)	−0.0012 (10)
C1	0.0205 (11)	0.0250 (11)	0.0342 (13)	−0.0001 (9)	0.0058 (9)	−0.0007 (10)
C2	0.0334 (14)	0.0362 (13)	0.0272 (13)	0.0006 (11)	0.0104 (10)	−0.0007 (10)
C3	0.0368 (14)	0.0371 (14)	0.0272 (13)	−0.0029 (11)	0.0006 (10)	−0.0058 (10)
C4	0.0252 (12)	0.0246 (11)	0.0300 (12)	0.0030 (9)	0.0088 (9)	0.0000 (9)
C5	0.0192 (11)	0.0197 (10)	0.0267 (11)	0.0029 (8)	0.0037 (8)	0.0000 (9)
C6	0.0202 (12)	0.0202 (11)	0.0286 (12)	−0.0009 (9)	0.0057 (9)	0.0007 (9)
C7	0.0232 (12)	0.0272 (11)	0.0252 (12)	0.0028 (9)	−0.0014 (9)	−0.0013 (9)
C8	0.0259 (12)	0.0303 (12)	0.0237 (12)	−0.0016 (10)	−0.0014 (9)	0.0030 (9)
C9	0.0176 (11)	0.0222 (10)	0.0270 (12)	−0.0015 (9)	0.0034 (9)	−0.0004 (9)
C10	0.0311 (13)	0.0344 (12)	0.0187 (11)	0.0057 (11)	0.0037 (9)	−0.0003 (9)

### Geometric parameters (Å, °)

**Table:**

S1—C9	1.729 (2)	C1—H1A	0.9300
S1—C8	1.814 (2)	C2—C3	1.376 (3)
O1—C6	1.214 (3)	C2—H2A	0.9300
N1—C4	1.338 (3)	C3—H3B	0.9300
N1—C3	1.342 (3)	C4—C5	1.390 (3)
N2—C9	1.379 (3)	C4—H4A	0.9300
N2—C6	1.405 (3)	C5—C6	1.488 (3)
N2—C7	1.479 (3)	C7—C8	1.510 (3)
N3—C9	1.295 (3)	C7—H7A	0.9700
N3—C10	1.335 (3)	C7—H7B	0.9700
N4—C10	1.150 (3)	C8—H8A	0.9700
C1—C2	1.383 (3)	C8—H8B	0.9700
C1—C5	1.391 (3)		
			
C9—S1—C8	92.34 (10)	C1—C5—C6	117.34 (19)
C4—N1—C3	116.86 (19)	O1—C6—N2	118.05 (19)
C9—N2—C6	128.16 (18)	O1—C6—C5	120.49 (19)
C9—N2—C7	112.40 (17)	N2—C6—C5	121.28 (18)
C6—N2—C7	117.80 (17)	N2—C7—C8	106.01 (17)
C9—N3—C10	118.32 (18)	N2—C7—H7A	110.5
C2—C1—C5	118.8 (2)	C8—C7—H7A	110.5
C2—C1—H1A	120.6	N2—C7—H7B	110.5
C5—C1—H1A	120.6	C8—C7—H7B	110.5
C3—C2—C1	118.5 (2)	H7A—C7—H7B	108.7
C3—C2—H2A	120.8	C7—C8—S1	104.14 (14)
C1—C2—H2A	120.8	C7—C8—H8A	110.9
N1—C3—C2	124.1 (2)	S1—C8—H8A	110.9
N1—C3—H3B	118.0	C7—C8—H8B	110.9
C2—C3—H3B	118.0	S1—C8—H8B	110.9
N1—C4—C5	123.4 (2)	H8A—C8—H8B	108.9
N1—C4—H4A	118.3	N3—C9—N2	122.30 (19)
C5—C4—H4A	118.3	N3—C9—S1	125.63 (17)
C4—C5—C1	118.4 (2)	N2—C9—S1	112.01 (15)
C4—C5—C6	123.50 (19)	N4—C10—N3	172.7 (2)
			
C5—C1—C2—C3	−1.3 (3)	C1—C5—C6—N2	150.65 (19)
C4—N1—C3—C2	0.6 (4)	C9—N2—C7—C8	34.3 (2)
C1—C2—C3—N1	0.2 (4)	C6—N2—C7—C8	−159.14 (18)
C3—N1—C4—C5	−0.3 (3)	N2—C7—C8—S1	−36.57 (19)
N1—C4—C5—C1	−0.8 (3)	C9—S1—C8—C7	25.54 (16)
N1—C4—C5—C6	−170.6 (2)	C10—N3—C9—N2	175.9 (2)
C2—C1—C5—C4	1.6 (3)	C10—N3—C9—S1	−7.2 (3)
C2—C1—C5—C6	172.0 (2)	C6—N2—C9—N3	−2.5 (3)
C9—N2—C6—O1	157.6 (2)	C7—N2—C9—N3	162.4 (2)
C7—N2—C6—O1	−6.6 (3)	C6—N2—C9—S1	−179.73 (17)
C9—N2—C6—C5	−27.3 (3)	C7—N2—C9—S1	−14.8 (2)
C7—N2—C6—C5	168.55 (18)	C8—S1—C9—N3	175.7 (2)
C4—C5—C6—O1	135.5 (2)	C8—S1—C9—N2	−7.10 (17)
C1—C5—C6—O1	−34.3 (3)	C9—N3—C10—N4	−178 (2)
C4—C5—C6—N2	−39.5 (3)		

### Hydrogen-bond geometry (Å, °)

**Table:**

*D*—H···*A*	*D*—H	H···*A*	*D*···*A*	*D*—H···*A*
C2—H2A···N4^i^	0.93	2.52	3.383 (3)	154
C8—H8B···N1^ii^	0.97	2.55	3.481 (3)	162

Symmetry codes: (i) *x*+1, −*y*+3/2, *z*−1/2; (ii) −*x*, −*y*+1, −*z*.
